# Human Serum Albumin Binds Streptolysin O (SLO) Toxin Produced by Group A *Streptococcus* and Inhibits Its Cytotoxic and Hemolytic Effects

**DOI:** 10.3389/fimmu.2020.507092

**Published:** 2020-12-08

**Authors:** Gian Marco Vita, Giovanna De Simone, Loris Leboffe, Francesca Montagnani, Davide Mariotti, Stefano Di Bella, Roberto Luzzati, Andrea Gori, Paolo Ascenzi, Alessandra di Masi

**Affiliations:** ^1^ Department of Sciences, Roma Tre University, Roma, Italy; ^2^ Department of Medical Biotechnologies, University of Siena, Siena, Italy; ^3^ Infectious and Tropical Diseases Unit, Department of Medical Sciences, Hospital of Siena, Siena, Italy; ^4^ Infectious Diseases Unit, Clinical Department of Medical, Siurgical, and Health Sciences, University of Trieste, Trieste, Italy; ^5^ Infectious Diseases Unit, Department of Internal Medicine, Fondazione IRCCS Ca’ Granda, Ospedale Maggiore Policlinico, Centre for Multidisciplinary Research in Health Science (MACH), University of Milan, Milan, Italy

**Keywords:** human serum albumin, red blood cells, streptolysin O, *Streptococcus pyogenes*, toxin

## Abstract

The pathogenicity of group A *Streptococcus* (GAS) is mediated by direct bacterial invasivity and toxin-associated damage. Among the extracellular products, the exotoxin streptolysin O (SLO) is produced by almost all GAS strains. SLO is a pore forming toxin (PFT) hemolitically active and extremely toxic *in vivo*. Recent evidence suggests that human serum albumin (HSA), the most abundant protein in plasma, is a player in the innate immunity “orchestra.” We previously demonstrated that HSA acts as a physiological buffer, partially neutralizing *Clostridioides difficile* toxins that reach the bloodstream after being produced in the colon. Here, we report the *in vitro* and *ex vivo* capability of HSA to neutralize the cytotoxic and hemolytic effects of SLO. HSA binds SLO with high affinity at a non-conventional site located in domain II, which was previously reported to interact also with *C. difficile* toxins. HSA:SLO recognition protects HEp-2 and A549 cells from cytotoxic effects and cell membrane permeabilization induced by SLO. Moreover, HSA inhibits the SLO-dependent hemolytic effect in red blood cells isolated from healthy human donors. The recognition of SLO by HSA may have a significant protective role in human serum and sustains the emerging hypothesis that HSA is an important constituent of the innate immunity system.

## Introduction

Group A *Streptococcus* (GAS, *Streptococcus pyogenes*) is a Gram-positive bacterium that causes a variety of diseases, ranging from pharyngitis to severe invasive infections, associated with a possible poor prognosis despite adequate antibiotic therapy. Its clinical impact is also related to the development of non-suppurative sequelae such as acute rheumatic heart fever and post-streptococcal glomerulonephritis (1). In the current therapy of severe streptococcal infection, a combination of clindamycin with a β-lactams molecule is used with the rationale to lower toxin production through protein synthesis reduction (clindamycin effect). Apart from a somatic constituent, a plethora of extracellular streptococcal toxins are involved in the pathogenesis of the severe invasive GAS diseases (2); thus, specific anti-toxin treatments are likely to have an impact on the clinical outcome. Among these extracellular products, the cytolysin streptolysin O (SLO) is produced by almost all GAS strains. SLO has a toxic activity toward red blood cells (RBCs), polymorphonuclear leukocytes, and platelets, as well as isolated mammalian and amphibian hearts. SLO has a tissue-destructive activity and is rapidly lethal when injected intravenously into mice or rabbits as a purified compound (3–5). At sublethal doses *in vivo*, SLO causes dermal necrosis, venous congestion, increased vascular permeability, and neurological abnormalities before death (6). More recently, SLO has been demonstrated to be essential in the pathogenesis of severe invasive infection in animal models (7).

SLO belongs to the cholesterol-dependent cytolysin (CDC) proteins, a conserved family of β-barrel pore-forming toxins (PFTs) that are secreted by Gram-positive bacteria (8, 9). PFTs are produced by many pathogenic bacteria from the genera *Escherichia*, *Staphylococcus*, *Clostridioides*, *Streptococcus*, *Listeria*, *Bacillus*, and *Arcanobacterium*, being important components of their virulence (10, 11). The CDCs share the undecapeptide and cholesterol binding motif, as well as the ability to form pores on host cell membranes *via* a cholesterol-dependent mechanism (9, 12). Although membrane cholesterol is required for the cytolytic mechanism of CDCs to form a complete transmembrane pore, also glycans receptors define the cellular tropism and are required for efficient deposition of PFTs, including CDCs, into cell membranes (8, 13–16). Following secretion from bacteria as soluble monomers, CDCs bind to host cell membranes through the *C*-terminal domain 4 (D4), undergo oligomerization, and form the pre-pore complex, which then collapses into the membrane to form the β-barrel pore that permeabilizes target cells and causes lysis and death (8, 17–20).

Human serum albumin (HSA) is the most abundant protein in plasma (∼7.0×10^−4^ M) and represents the main modulator of fluid distribution between body compartments. By acting as a depot and carrier for many endogenous and exogenous compounds, HSA affects the pharmacokinetics of many drugs, and accounts for most of the antioxidant capacity of human serum (21–24). HSA is a 66 kDa all-α-helical protein built by three homologous domains (labeled I, II, and III). Fatty acids (FAs) represent the primary physiological ligands of HSA (24). HSA acts as a self-defense mechanism toward *Clostridioides difficile* (*C. difficile*) infection (CDI) by binding both toxins A (TcdA) and B (TcdB); indeed, HSA induces a conformational change that promotes toxins autoproteolysis outside the intestinal epithelial cells. This event prevents toxins internalization and the consequent cytotoxic and cytopathic effects, both *in vitro* and *in vivo*. This provides an explanation for the clinical correlation between CDI severity and hypoalbuminemia (25, 26).

Here, the capability of HSA to bind SLO and to modulate its pathogenic actions is reported by combined *in silico*, *in vitro*, and *ex vivo* approaches. We showed that HSA binds SLO with high affinity through a non-conventional binding site located in domain II. This binding inhibits SLO cytotoxicity *in vitro* in human epithelial type 2 (HEp-2) and human lung carcinoma (A549) cells and significantly reduces its hemolytic effects in red blood cells. Overall, these results support the assumption that HSA protects human cells from SLO-dependent toxicogenic effects.

## Materials and Methods

### Ethical Standards

Human RBCs were collected from four of the co-authors of the present work, who voluntarily, without constraints, and freely donated blood samples for the study purposes. Therefore, Institutional Review Board approval was not requested. Blood has been collected in accordance with the World Medical Association’s Declaration of Helsinki.

### Commercial Proteins

SLO isolated from GAS (S5265; Merck KGaA, Darmstadt, Germany) was dissolved in cold deionized water to obtain a stock solution of at least 125 U/μL (according to the manufacturer’s units indication), corresponding to 2.2×10^−6^ M. SLO has been always activated by incubation with 2.0×10^−2^ M dithiothreitol (DTT; Merck KGaA) for 15 minutes at 37°C. Fatty acid- and globulin-free HSA (99.5%; A3782; Merck KGaA) and fatty acid-free bovine serum albumin (BSA) (A4612; Merck KGaA) were dissolved in deionized water at a final concentration of 2.0×10^−3^ M, corresponding to 132 mg/mL. All commercial proteins were of reagent grade and used without further purification. SLO, HSA, and BSA concentrations were determined spectrophotometrically using the following values of ε_280nm_: 71.280, 39.310, and 43.824 M^−1^ cm^−1^, respectively (http://www.expasy.org). SLO, HSA, and BSA were handled following the manufacturer’s instruction and using established good laboratory practices under biosafety cabinets with installed HEPA filters to avoid lipid contamination.

### Cloning, Expression, and Purification of Recombinant Wild-Type and Double Mutant Leu305Ala/Phe374Ala HSA

The pPICZaB vector containing the HSA transcript (Sequence ID: NM_000477.7) was used to amplify by PCR the *Alb* gene using the following primers: FW-HindIII_NheI: 5’-caagcttgctagcgatgcacacaagagtgaggtt-3’ and RV-BamHI: 5’-cgcggatccttataagcctaagg-3’. The HSA amplicon of 1758 base pairs (bps) was first sub-cloned in the pBluescript KS(−) plasmid and then cloned into the pET-28a (+) vector. The double mutant Leu305Ala/Phe374Ala (hereafter named L305A/F374A) was generated using the QuikChange Lightning Site-Directed Mutagenesis Kit (Agilent Technologies, Santa Clara, CA, USA). The pET-28a(+)-HSA was used as a template and oligonucleotides were designed using the QuikChange Primer Design program provided by Agilent Technologies (http://www.genomics.agilent.com/primerDesignProgram.jsp). The following primers were used to produce, by a two-step PCR, the double mutant: FW-L305A: 5’-tgagatgcctgctgacttgccttcagcagctgctgatttt-3’; RV-L305A: 5’-cttcatagtggaccaacttcaaatcccctcctttgg-3’; and FW-F374A: 5’-ctcatgaatgctatgccaaagtggccgatgaatttaaacctcttgtgg-3’; RV-F374A: 5’-ccacaagaggtttaaattcatcggccactttggcatagcattcatgag-3’. The DNA sequence of the double mutant was verified by DNA sequencing (**Figure S1A**). The Origami (DE3) strain (Novagen; Darmstadt, Germany) was used to express the recombinant wild-type 6×His-tag-HSA (hereafter named wt-HSA) and the L305A/F374A 6×His-tag-HSA (hereafter named L305A/F374A-HSA) by the auto-induction system. Briefly, bacteria were auto-induced in NZY broth (NZYTech, Lisbon, Portugal) when OD_600_ = 1, for 12 h at 25°C and 18°C for wt-HSA and L305A/F374A-HSA, respectively. The bacterial pellet was lysed in 2.0×10^−2^ M phosphate buffer pH 7.4, 1.4×10^−1^ M NaCl, 10% (*v/v*) trehalose and 0.015% (*v*/*v*) Tween-20 (Merck KGaA). Then, the supernatant was loaded onto a His-Trap affinity chromatography column (GE Healthcare Bio-Sciences, Uppsala, Sweden). The adsorbed wt-HSA was eluted by a linear gradient of imidazole (2.0×10^−2^ M phosphate buffer pH 7.4, 5.0×10^−1^ M NaCl, and 1.0×10^−2^ to 1.0 M imidazole). The fractions containing either the wt-HSA or the L305A/F374A-HSA were dialyzed against 2.0×10^−2^ M phosphate buffer pH 7.0 and 1.5×10^−1^ M NaCl, and subsequently loaded onto a Superdex™ 75 10/300 GL column. The eluate was analyzed by Coomassie staining and Western blot (**Figure S1B**). Wt-HSA and L305A/F374A-HSA concentrations were determined spectrophotometrically using a value of ε_280nm_ of 39.310 M^−1^ cm^−1^ (http://www.expasy.org). Lipid-free HSA was prepared according to the charcoal treatment (27). Recombinant wt- and L305A/F374A-HSA were handled using established good laboratory practices under biosafety cabinets with installed HEPA filters to avoid lipid contamination.

### Protein-Protein Docking

Docking simulations of the SLO three-dimensional structure (PDB ID: 4HSC) bound to HSA (PDB ID: 1AO6) were performed using ZDOCK 3.0.2 server (28). ZDOCK implements a Fast Fourier Transform algorithm and a scoring system based on a combination of shape complementarity, electrostatics, and statistical potential terms. The top 216 docked structures for the SLO-HSA complex were rescored using ZRANK (29), which uses a more detailed potential including electrostatics, van der Waals, and desolvation terms. The best pose was finally refined by Rosetta Docking 2 server that uses the Rosetta force field to evaluate the energy of the complexes (30).

### Spectrofluorimetric Binding Assay

Values of the apparent dissociation equilibrium constant for binding of SLO to the recombinant wt-HSA or to the L305A/F374A-HSA (i.e., *K*_d_) were determined by mixing the toxin solution (final concentration 2.0×10^−8^ M) with the HSA solution (final concentration ranging from 0 M to 4.0×10^−8^ M). The formation of the HSA:SLO complex was monitored spectrofluorimetrically between 300 nm and 400 nm. The excitation wavelength was 280 mm and the slit width was 5 nm. Values of *K*_d_ were obtained from the dependence of the fluorescence intensity change (*ΔF*) on the HSA concentration (i.e., [HSA]), according to Eq. 1:

(1) $$\Delta F=\Delta F_{\text{tot}}-((\Delta F_{\text{tot}}\times [\text{HSA}])/(K_{\text{d}}+[\text{HSA}]))$$

where *ΔF*_tot_ is the total fluorimetric change.

### ELISA Binding Assay

The amount of HSA required to efficiently coat wells of the ELISA plate (NUNC MaxiSorp™ flat-bottom, Thermo Fisher Scientific, Waltham, MA, USA) was determined using 1.0×10^−8^ M, 4.0×10^−8^ M, 8.0×10^−8^ M, and 1.6×10^−7^ M commercial HSA (**Figure S2**). On the basis of these preliminary experiments, wells were coated overnight (O/N) at 4°C with 100 μL of 1.6×10^−7^ M commercial HSA, washed three times in Phosphate Buffer Saline pH 7.4 (PBS; 3.7×10^−2^ M NaCl, 1.0×10^−2^ M phosphate, 2.7×10^−3^ M KCl) (Merck KGaA), and finally blocked in 5% milk (*w*/*v*) (Bio-Rad, Hercules, CA, USA) dissolved in PBS/0.05% Tween-20 (*v*/*v*) (PBS/T) for 1 h at room temperature (RT). After three washes in PBS/T, HSA-coated wells were incubated with 1.0×10^−8^ M and 2.0×10^−8^ M of activated SLO diluted in 3% nonfat dry milk dissolved PBS/T for 1 h and 30 min at RT. After plates washing in PBS/T, SLO bound to HSA was detected using the polyclonal anti-SLO primary antibody (ab188539, Abcam, dilution 1:2000) and the goat anti-rabbit secondary antibody (#1706515, Bio-Rad, dilution 1:5000). Finally, 100 μL of tetramethylbenzidine (TMB; Merck KGaA) was added to each well and the colorimetric reaction was measured at 370 nm until reaction saturation using the Tecan Spark 10M (Tecan, Männedorf, Switzerland). Microtiter wells coated with 100 μL of 1.6×10^−7^ M commercial HSA, without the subsequent addition of SLO, were used as a negative control.

### Pull-Down Binding Assay

Fifteen μL of magnetic beads (MagneHis Protein Purification System, Promega; Wisconsin, USA) were added to 4 μg of recombinant wt-HSA resuspended in 2.0×10^−2^ M phosphate buffer pH 7.4 (1.6×10^−2^ M Na_2_HPO_4_·7 H_2_O, 3.6×10^−4^ M NaH_2_PO_4_·H_2_O, 5.0×10^−1^ M NaCl) (Merck KGaA) and incubated 15 min at RT in slow rotation. After washing beads three times with phosphate buffer pH 7.4, 1.5 μg of activated SLO was added to the beads and incubated 1 h and 30 min at RT in slow rotation. The unbound fraction was removed, and beads were washed 3 times in phosphate buffer pH 7.4. Finally, the elution buffer containing imidazole was used to detach proteins from the beads. The presence of SLO in the elution fractions was revealed by Western blot. The cross-reactivity of the anti-SLO antibody was tested (**Figure S3**).

### Western Blot

SLO (45, 90, 135, and 270 ng, corresponding to 3%, 6%, 9%, and 18% of 1.5 μg SLO used in the magnetic beads assay, hereafter named input) was loaded on a 12.5% SDS-PAGE together with 30 microliters of magnetic beads eluates (half volume of the entire eluates). After blotting on polyvinylidene ﬂuoride (PVDF) membranes (Bio-Rad), filters were blocked for 1 h at RT with either 3% nonfat dry milk (*w*/*v*)/0.1% Tween-20 (*v*/*v*) in TBS or 3% BSA (*w*/*v*)/0.1% Tween-20 (*v*/*v*) in TBS (*w*/*v*). Membranes were incubated O/N at 4°C with anti-SLO (ab188539; Abcam, dilution 1:4000) or anti-HSA (sc-50535; Santa Cruz Biotechnology, dilution 1:200) primary antibodies, and finally incubated for 1 h at RT with a goat anti-rabbit IgG secondary antibody (#1706515, Bio-Rad, dilution 1:10000). Proteins were visualized using the Clarity™ Western ECL substrate (Bio-Rad). Images were acquired using the ChemiDoc™ Imaging system (Bio-Rad). Protein levels were quantified using the Image Lab software (version 2.1.0.35.deb, Bio-Rad).

### Cell Line and Culture Condition

Human laryngeal carcinoma epithelial type-2 cells (HEp-2) were cultured in Eagle’s minimal essential medium (EMEM) (Corning, VA, USA), whereas A549 human lung carcinoma epithelial cells were cultured in Dulbecco’s Modified Eagle Medium (DMEM) (Corning). Both mediums were complemented with 10% fetal bovine serum (FBS) (BioWest, Nuaillé, France), 1% (*v*/*v*) nonessential amino acids (Biochrome GmbH, Berlin, Germany), 100 μg/mL penicillin and streptomycin (Merck KGaA), and 2.0×10^−3^ M L-glutamine (Merck KGaA). Cells were grown at 37°C and 5% CO_2_. Cells were counted using the BLAUBRAND^®^ counting chamber (Brand GMBH, Wertheim, Germany).

### MTT Assay

For the determination of IC50 values, dose-response (75, 150, 300, 600, and 1000 U/mL SLO) and time-course (0.5, 3, 6, and 24 h) experiments were performed. Briefly, HEp-2 and A549 were seeded in flat-bottomed 96-well plates at a concentration of 1.5×10^4^ cells/well. After 24 h, confluent cell monolayers were placed in serum-free medium and treated with increasing concentrations of activated SLO. To evaluate the effect of HSA, HEp-2 and A549 cells were treated with 600 U/mL of activated SLO for 0.5, 6, and 24 h, in the absence or presence of 1.0×10^−5^ M and 1.0×10^−4^ M HSA. Cell viability was measured by the 3-(4,5-dimethylthiazol-2-yl)-2,5-diphenyltetrazolium bromide solution (MTT; 0.5 mg/mL stock solution; Merck KGaA), which was added to the cell culture and incubated for 4 h at 37°C. Formazan crystals were then dissolved in lysis buffer (4.0×10^−3^ HCl, 0.1% Triton X-100 [*v*/*v*] in isopropanol). Plates were analyzed using a microplate reader at 570 nm (Victor 2, Perkin Elmer, Hopkinton, MA). As SLO was dissolved in deionized water and activated with DTT, this solvent was used as a control vehicle in untreated cells adding the same volume used in SLO-treated cells.

### Cell Membrane Labeling With Fluorescent Dye

A suspension of 2.0×10^7^ HEp-2 cells was stained with the PKH67 fluorescent cell linker dye solution (Merck KGaA) following the manufacturer’s instruction. Once stained, 2.5×10^6^ HEp-2 cells were seeded on coverslips in a FBS-deprived medium. After 24 h, cells were treated for 4 minutes at 37°C with 100 U/mL activated SLO in the absence or presence of 1.0×10^−4^ M HSA resuspended in Ringer’s solution (1.6×10^−1^ M NaCl, 3.0×10^−3^ M KCl, 2.0×10^−3^ M CaCl_2_, 1.0×10^−3^ M MgCl_2_, 3.0×10^−3^ M NaH_2_PO_4_, 5.0×10^−3^ M HEPES pH 7.2, and 1.0×10^−2^ M glucose). The incubation was stopped by adding complete EMEM medium to cells. In order to detect dead cells, a solution of 20 μg/mL propidium iodide (PI; Merck KGaA) was added to unfixed cells. Cells were immediately analyzed and images acquired by confocal microscopy (LCS Leica confocal microscope; Leica Microsystems,Heidelberg, Germany).

### Ex Vivo Red Blood Cell Hemolysis Assay

The RBC cell hemolysis assay was performed according to previously published protocols (8, 31). Briefly, human whole blood was centrifuged at 500×g for 5 min, in order to separate the RBCs from plasma. The pellet was washed three times in PBS and diluted in PBS pH 7.4 to reach an RBCs final concentration of 2% (*v*/*v*). As reported on the manufacturer’s instruction, 1 U of activated SLO cause a 50% lysis of 50 μL of 2% human RBCs suspensions in PBS at 37°C. Therefore, after seeding 190 μL of 2% RBCs solution into a V-bottom 96-well plate, a dose-response curve was performed by adding 1, 2.5, and 5 U activated SLO for 1 h at 37°C. To evaluate the protective effect of HSA, at least 5 U activated SLO were added to 2% RBCs solution, in the absence or presence of 1.0×10^−5^ M and 1.0×10^−4^ M HSA or 1.0×10^−4^ M bovine serum albumin (BSA). As a positive control, RBCs were lysed by adding 2% (*v*/*v*) Triton X-100. As a negative control, RBCs were incubated with PBS. Following the incubation, cells were centrifuged at 500×g for 8 min and the supernatant was transferred into a flat-bottomed 96-well plate. The concentration of released hemoglobin was quantiﬁed by measuring the absorbance at 541 nm (ε_HbO2_ = 13.8 M^−1^ cm^−1^;[32]) using a plate reader (EL800 Microplate Reader, BioTeK Instruments, Winooski, VT, USA). Results were represented as the percentage of lysed RBCs, assuming as 100% the positive control. RBCs were isolated from four healthy donors.

### Data Analysis

Results are shown as mean ± standard deviation (SD) derived minimally from 3 independent experiments. Statistical significance between means, assessed by either One-way or Two-way ANOVA (GraphPad InStat 3.1 Software Inc., San Diego, CA, USA), was considered significant when P values were ≤ 0.05. For survival assays expressed as percentages, arcsin transformations were performed prior to ANOVA analysis.

## Results

### Molecular Docking of SLO Binding to HSA

**Figure 1:**
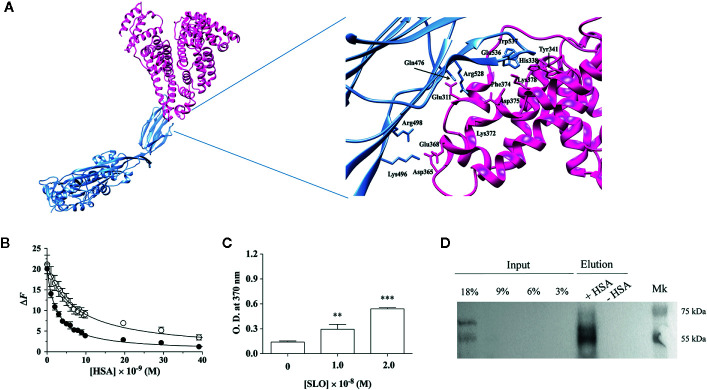
HSA binds SLO toxin produced by *Streptococcus pyogenes*. **(A)** Molecular docking of SLO binding to HSA. Left: overall view of the putative complex between HSA (PDB ID: 1AO6) (deep pink) and SLO (PDB ID: 4HSC) (light blue) obtained by docking simulations. Right: residues involved in HSA-SLO interactions are highlighted. Images were drawn with the UCSF Chimera-package (35). **(B)** SLO binding to wt-HSA (filled circles) and to L305A/F374A-HSA (open circles) evaluated by spectrofluorimetric analysis. The continuous lines were calculated according to Eqn. 1 with the following parameters: wt-HSA - *ΔF*_tot_ = 20.1 and *K*_d_ = (2.5 ± 0.2)×10^−9^ M; and L305A/F374A-HSA - *ΔF*_tot_ = 21.1 and *K*_d_ = (7.3 ± 0.5)×10^−9^ M. Where not shown, the error bar is smaller than the symbol. **(C)** SLO binding to HSA evaluated by ELISA. Either 1.0×10^−8^ M or 2.0×10^−8^ M of activated SLO were added to HSA-coated wells, and the detection of HSA:SLO interaction was performed after incubation with anti-SLO antibody. Readings were performed using the TMB colorimetric substrate at 370 nm until reaction saturation. Microtiter wells coated with 100 μL of 1.6×10^−7^ M commercial HSA, without the subsequent addition of SLO, were used as a negative control. Results are representative of triplicates and are expressed as mean ± SD (One-way ANOVA, **P < 0.01 and ***P < 0.001). **(D)** SLO binding to recombinant wt-HSA evaluated by magnetic beads assay. Activated SLO (2.9×10^−7^ M) was incubated with 4 μg of wt-HSA-conjugated (+) or unconjugated (−) beads. Thirty microliters of magnetic beads eluates were resolved on a 12.5% SDS-PAGE and SLO bound to HSA was detected by immunoblot using anti-SLO antibody. Different quantities of SLO (i.e., 45, 90, 135, and 270 ng corresponding to 3%, 6%, 9%, and 18% of 1.5 μg SLO) were loaded as inputs. Results show the presence of the bands corresponding to SLO in the eluates.

SLO consists of four domains (D1–D4), among which D3 provides the transmembrane spanning regions of the toxin and D4, which contains the highly conserved undecapeptide sequence (Glu529-Arg539), takes part in the initial interactions with the membrane, including direct recognition of cholesterol (18, 32–34). The SLO residues Gln476, Trp503, Trp537, and Trp538, of which Trp537 and Trp538 are located within the undecapeptide, play a key role in the recognition and binding to the host cell membrane (8, 19). The docking analyses predicted that the interaction between SLO and HSA involves the D4 of SLO and the domain II of HSA (**Figure 1A**). In particular, the binding interface is lined by residues Leu480, Trp503, Ala534, Trp537, and Trp538 of SLO, and residues Leu305, Ala306, Val310, Tyr341, Phe374, Phe377, and Val381 of HSA. These residues are located nearby each other, suggesting a hydrophobic nature of the interaction. Remarkably, HSA residues involved in the interaction with SLO are the same as those involved in the recognition of *Clostridioides difficile* toxins (26). The HSA-SLO complex is stabilized by a hydrogen bond between Glu311 and Lys378 residues of HSA and Gln476 and Glu536 side chains of SLO, respectively. Interestingly, Trp503, Trp537, and Trp538 of SLO involved in the recognition of HSA are located in the undecapeptide region, which is a signature sequence in CDCs that plays a key role in the interaction with host membrane cholesterol (8).

### SLO Binding to HSA

On the basis of docking results, Leu305 and Phe374 residues of HSA were mutated to evaluate their role in SLO recognition. We showed that recombinant wt-HSA and L305A/F374A-HSA bind to SLO in a saturating dose-dependent manner (**Figure 1B**). According to Eqn. 1, data analysis indicated that the affinity of wt-HSA for SLO (*K*_d_ = [2.5 ± 0.2]×10^−9^ M) is higher than that of L305A/F374A-HSA (*K*_d_ = [7.3 ± 0.5]×10^−9^ M).

SLO binding to HSA was confirmed by ELISA assay that was performed by coating wells with HSA (**Figure S2**) and testing SLO binding. Results obtained indicated a dose-dependent increase of the absorbance values, with a 2-fold (*P*<0.01) and a 3.8-fold (*P*<0.001) increase at 1.0×10^−8^ M and 2.0×10^−8^ M SLO compared to HSA-coated well without SLO addition (i.e., 0 M SLO) (**Figure 1C**).

Pull-down experiments using recombinant wt-HSA bound to Ni^2+^-magnetic beads further supported HSA:SLO interaction as revealed by the presence of the 69 and 55 kDa bands of SLO (36) in the elution fraction (**Figure 1D**, lane + HSA). To confirm wt-HSA binding to the beads, the presence of HSA was detected by Western blot (**Figure S4**).

### HSA Exerts a Neutralizing Effect Toward SLO-Induced Cytotoxicity in Epithelial HEp-2 and A549 Cells

The cytotoxic activity of SLO *in vitro* is significantly increased in the presence of reducing agents (e.g., DTT) (36, 37). To achieve SLO activation without inducing cytotoxic effects, preliminary experiments were performed using HEp-2 and A549 cells to find out the optimal concentration of DTT to be used. Results showed that 2.0×10^−3^ M DTT was sufficient to induce SLO activation without affecting cell viability (**Figure S5**).

**Figure 2:**
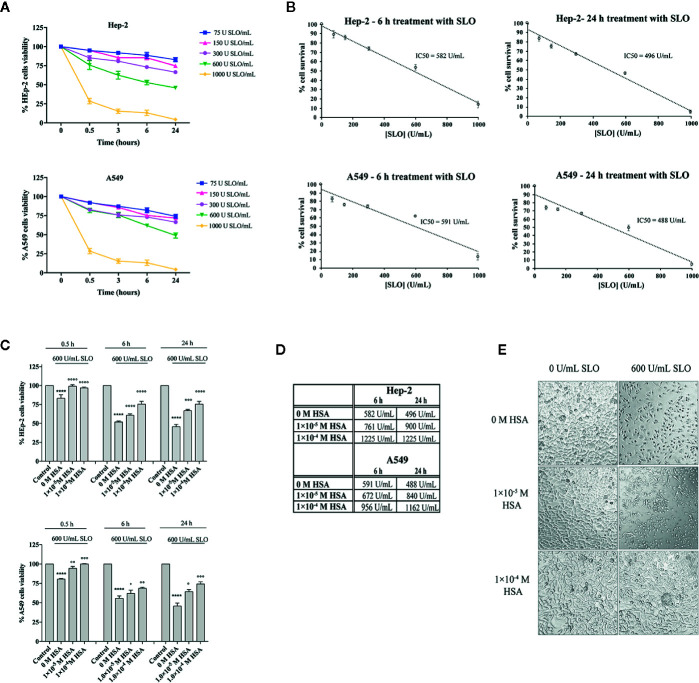
HSA protects human HEp-2 cells from intoxication with SLO toxin. **(A)** Dose- and time-course experiments to evaluate SLO cytotoxic effects in HEp-2 and A549 cells. Cells were treated for 0.5, 3, 6, and 24 h with 75, 150, 300, 600, and 1000 U/mL of activated SLO. The percentage of viable cells was determined by the MTT metabolic test, considering that untreated cells were taken as 100%. Data represent the mean value ± SD derived from three replicates. **(B)** IC50 values were calculated by plotting cell survival (%) versus SLO concentration (U/mL) in HEp-2 and A549. **(C)** HEp-2 cells and A549 cells were treated for 0.5, 6, and 24 h with 600 U/mL of activated SLO, in the absence or presence of 1.0×10^−5^ M and 1.0×10^−4^ M HSA. The percentage of viable cells was determined by MTT test, considering that untreated cells (Control) were taken as 100%. Data represent the mean value ± SD derived from three replicates (Two-way ANOVA and Tukey’s *post hoc* test [****P < 0.0001 compared with control; °*P* < 0.05, °°*P* < 0.01, °°°*P* < 0.001, and °°°°*P* < 0.0001 compared with cells treated with 600 U/mL SLO in the absence of HSA]). **(D)** IC50 values were calculated assuming a linear fit in HSA-treated Hep-2 and A549 cells. **(E)** Exemplificative images show the morphologies of HEp-2 cells untreated (i.e., 0 U/mL SLO and 0 M HSA) and treated for 24 h with 600 U/mL of activated SLO in the absence or presence of 1.0×10^−5^ M and 1.0×10^−4^ M HSA. Images were acquired using a Leica microscope (Leica Microsystems, Heidelberg, Germany); magnification 20×.

Next, a dose-response and time-course analysis of SLO cytotoxicity in HEp-2 and A549 cells grown in the FBS-deprived medium was performed. According to literature (38), cells treatment with SLO caused a dose- and time-dependent decrease in cell survival (**Figure 2A**). On the basis of the IC50 values calculated (**Figure 2B**), we treated HEp-2 and A549 cells with 600 U/mL SLO for 0.5, 6, and 24 h. HEp-2 cells pre-incubated with tolerated doses of HSA (**Figure S6**) before exposure to 600 U/mL SLO for 0.5 h showed a cell viability increase of ~15% at 1.0×10^−5^ M and 1.0×10^−4^ M HSA (*P*<0.0001; IC50 >5000 U/mL), compared to HSA-untreated cells (IC50 = 582 U/mL) (**Figures 2C, D**). HEp-2 cells treated for 6 h with 600 U/mL SLO showed a 15% cell viability increase in the presence of 1.0×10^−5^ M HSA (*P*<0.0001; IC50 = 761 U/mL) and a 30% increase in the presence of 1.0×10^−4^ M HSA (*P*<0.0001; IC50 = 1225 U/mL), compared to HSA-untreated cells (IC50 = 582 U/mL) (**Figures 2C, D**). Finally, HEp-2 cells treated for 24 h showed a cell viability increase of 12% in the presence of 1.0×10^−5^ M HSA (*P*<0.001; IC50 = 900 U/mL) and 21% in the presence of 1.0×10^−4^ M HSA (*P*<0.0001; IC50 = 1225 U/mL), compared to HSA-untreated cells (IC50 = 496 U/mL) (**Figures 2C, D**). Conversely, A549 cells treated with 600 U/mL SLO for 0.5 h showed a cell viability increase of ~14% (*P*<0.01; IC50 > 5000 U/mL) and 20% (*P*<0.001; IC50 > 5000 U/mL) in the presence of 1.0×10^−5^ M and 1.0×10^−4^ M HSA, respectively, compared to HSA-untreated cells (IC50 = 582 U/mL) (**Figures 2C, D**). The 6 h treatment caused a 20% increase of cell viability only in the presence of 1.0×10^−4^ M HSA (*P*<0.01; IC50 = 956 U/mL), whereas the 24 h treatment indicated that cell viability increased up to 10% (*P*<0.05; IC50 = 840 U/mL) and 20% (*P*<0.001; IC50 = 1162 U/mL) at 1.0×10^−5^ M and 1.0×10^−4^ M HSA, respectively, compared to HSA-untreated cells (IC50 = 488 U/mL) (**Figures 2C, D**). The protective effects of HSA were also evident when cells were examined microscopically, as they showed a condensed nuclear morphology compared to noninfected cells that showed a normal healthy appearance with no signs of chromatin condensation (**Figure 2E**).

**Figure 3:**
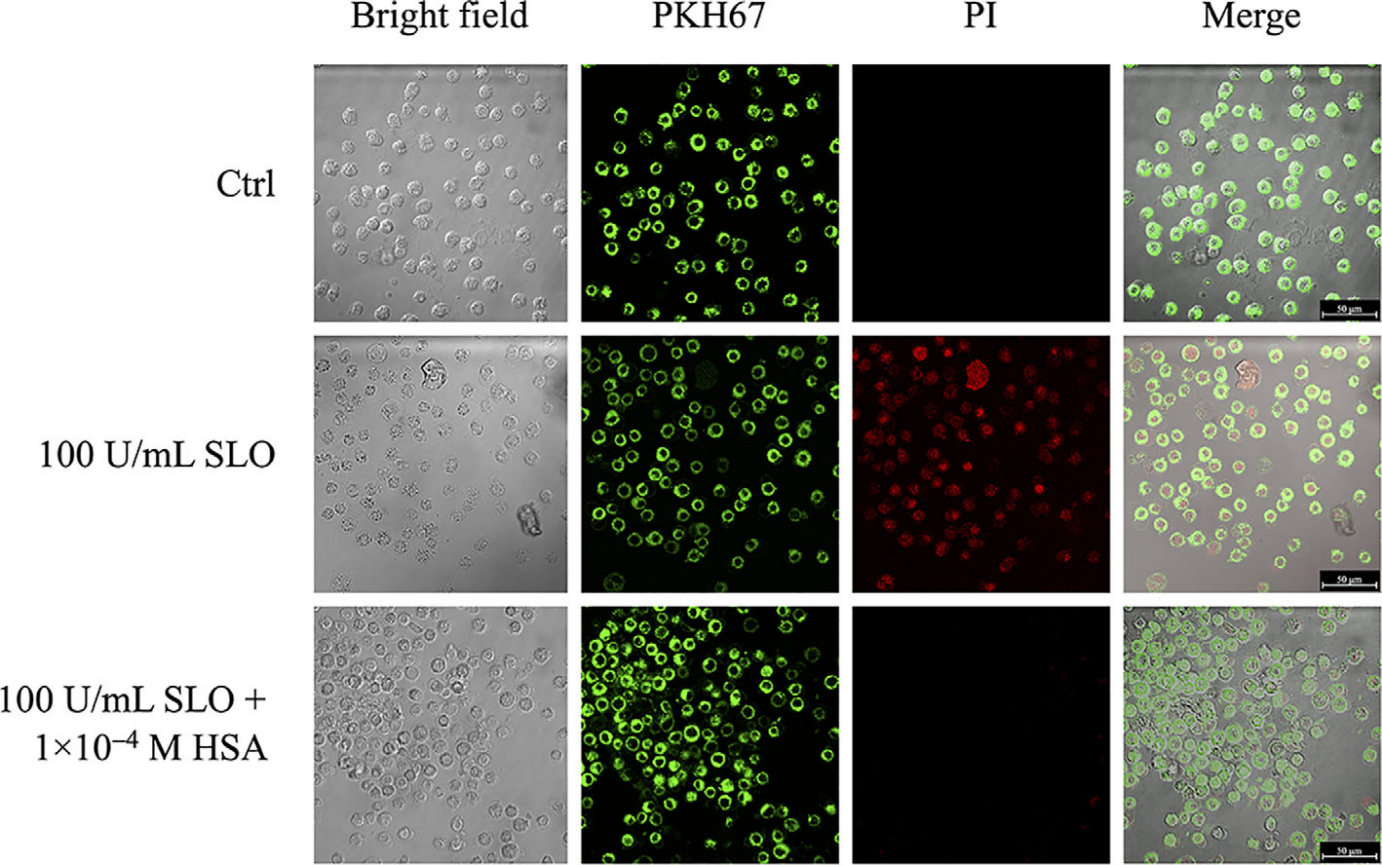
HSA inhibits SLO-induced cells membrane permeabilization. HEp-2 cells were stained with the PKH67 fluorescent cell linker dye solution (green signal), in order to visualize cells membrane. After 24 h, stained cells were treated with 100 U/mL of activated SLO, in the absence or presence of 1.0×10^−4^ M HSA. Propidium iodide (PI) staining, which is not permeant to live cells, allowed to detect dead cells. Cells were immediately analyzed and acquired using the LCS Leica confocal microscope (Leica Microsystems, Heidelberg, Germany).

As the docking analysis revealed that HSA recognizes part of the undecapeptide region of SLO (**Figure 1**), which is required to bind membrane cholesterol of the host cells and to exert SLO cytolytic activity (8), we studied whether HSA was able to protect HEp-2 cells from SLO-dependent permeabilization. Cells staining with the PKH67 fluorescent cell linker dye solution allowed visualization of cell membranes, whereas propidium iodide (PI) staining, which is not permeant to live cells, binds the nucleic DNA of permeabilized dead cells. Results obtained showed that SLO induced cell permeabilization (100% PI-positive cells), whereas the presence of HSA in the medium completely inhibited the SLO-dependent permeabilization (**Figure 3**).

### HSA Protects From SLO-Induced Cytotoxicity in Red Blood Cells

**Figure 4:**
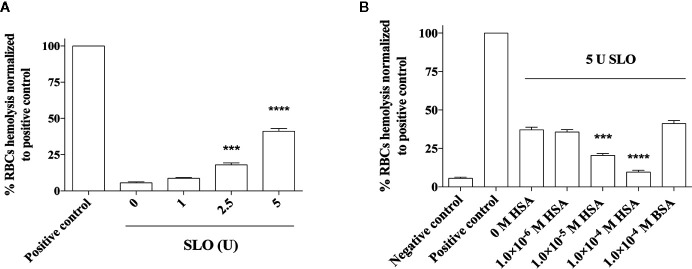
Effect of HSA and BSA on the SLO toxin-mediated red blood cells (RBCs) hemolysis. RBCs were isolated from the human whole blood of four healthy donors and treated with **(A)** 0, 1, 2.5 and 5 U of activated SLO for 1 h at 37°C. **(B)** Considering that 1 U of activated SLO causes a 50% lysis of 50 μL of 2% human RBCs, we used 5 U of activated SLO to cause the 50% lysis of 190 mL of 2% human RBCs. RBCs were treated with the toxin for 1 h at 37°C, in the absence or presence of 1.0×10^−6^ M to 1.0×10^−4^ M HSA. The protective effect of 1.0×10^−4^ M BSA was tested. As negative control, RBCs were incubated with PBS, in the absence of activated SLO. As positive control, RBCs were disrupted with 2% Triton X-100. The concentration of released hemoglobin was quantiﬁed by measuring the absorbance at 541 nm. Results were represented as the percentage of lysed RBCs (assuming as 100% the positive control) derived from three independent experiments ± SD (One-way ANOVA, ***P < 0.001 and ****P < 0.0001 compared with cells treated with 600 U of activated SLO in the absence of HSA).

The protective effect of HSA toward SLO-induced cytotoxicity was also evaluated *ex vivo* by a hemolytic assay (**Figure 4**). First, a dose-response analysis of SLO hemolytic effect in RBCs was performed (**Figure 4A**). Results obtained showed a percentage of hemolysis proportional to the units of SLO added, with a 40% hemolysis in RBCs treated with 5 U SLO compared to the negative control. The pre-incubation of SLO for 15 minutes with either 1.0×10^−5^ M or 1.0×10^−4^ M HSA caused a reduction of RBCs hemolysis to 20% (*P*<0.001) and 9% (*P*<0.0001), respectively, compared to HSA-untreated cells exposed to SLO. However, 1.0×10^−4^ M BSA did not inhibit the SLO-dependent hemolysis indicating that the neutralizing effect is specific for human albumin (**Figure 4B**). This is not unusual; indeed, it has been reported that the binding sites of human, bovine, equine, and leporine albumin are not conserved and do not always coincide (39). Of note, HSA alone did not exert any hemolytic effect at both concentrations tested (1.0×10^−5^ M and 1.0×10^−4^ M) (**Figure S7**).

## Discussion

In the last years, the hypothesis that HSA is a player in the innate immunity “orchestra” has emerged (25, 26, 40). Previously, we demonstrated that HSA binds *C. difficile* TcdA and TcdB toxins that reach the bloodstream after being produced in the colon (25, 26). Here, we report the *in vitro* and *ex vivo* capability of HSA to neutralize the effects of the PFT produced by GAS, i.e., SLO. HSA binds SLO with high affinity through a non-conventional binding site located in domain II. This interaction, involving the same domain of HSA that has been previously demonstrated to interact with the TcdA and TcdB toxins of *C. difficile* (26), protects epithelial HEp-2 and A549 cells as well as *ex vivo* RBCs from the cytotoxic effects of SLO. The HSA:SLO interaction takes place outside cells impairing the SLO-dependent permeabilization of cell membranes.

Human cells put in place several protective mechanisms against PFTs such as changes in membranes receptors, membranes repair, activation of cell stress responses, and increased nutrient (e.g., glutamine) demand for immune cells metabolism (10, 41–46). Results here reported contribute in defining the relevance of HSA in human physiology and pathophysiology that goes far behind its major role of regulator of fluid distribution in body compartments. Indeed, HSA is capable to bind and inhibit the hemolytic activity of the SLO toxin produced by GAS by binding to domain II. Mutagenesis experiments indicate a direct interaction between SLO and HSA, as the replacement of Leu305 and Phe374 residues located in the binding interface of HSA caused a decrease in the affinity for SLO. This non-canonical binding site seems to be specifically devoted to the recognition of toxins and proteins expressed by bacteria. Several Gram-positive bacterial species, including human pathogens, express surface proteins that interact with host proteins like HSA and IgG with high specificity and affinity (24, 47). In 1979, Kronvall and coworkers first described the binding of HSA to bacterial surface structures and found that groups A, C, and G streptococci specifically absorbed HSA from plasma (48). Subsequently, some strains of *Finegoldia magna* were also found to bind HSA. In the case of group C and G streptococci, protein G is responsible for HSA binding. Some isolates of *F. magna* bind HSA to their surface; the molecule responsible for the HSA:*F. magna* complexation is called poly(A)-binding protein (PAB) (49), which is the homologue of streptococci protein G (50, 51). Protein G and PAB share the GA module whose exact function is unknown (50, 51). The crystal structure of HSA in complex with the GA module of *F. magna* PAB highlighted that the interaction involves domain II of HSA (52). However, differently from the protective effects of HSA toward *C. difficile* TcdA and TcdB (25, 26) and *S. pyogenes* SLO toxins (data here reported), binding of HSA to the GA module of *F. magna* could provide growing bacteria with FAs and, possibly, other nutrients transported by HSA (52, 53). Overall, the selective recognition of bacteria proteins and toxins through domain II of HSA represents a clear example of host-microbe adaptation at the molecular level.

Our work demonstrates that HSA protects from SLO-induced cytotoxicity. Since this effect is the result of the extracellular binding between HSA and SLO, from a clinical point of view the demonstration of a neutralizing effect itself rather than the protective effect on different types of adopted cellular models is the most relevant result. *In vivo* studies clearly demonstrated that the inhibition of SLO activity was significantly associated with a disease severity reduction in necrotizing fasciitis, necrotizing myositis, bacteremia, and soft tissue infection models (54, 55). Therefore, we hypothesize that HSA levels could affect streptococcal diseases severity.

In conclusion, here for the first time the interaction between HSA and SLO exotoxin has been reported. Even if further studies are needed to confirm *in vivo* interaction, we demonstrated that HSA is able to partially inhibit the cytolytic and hemolytic activity of SLO, which are the two main toxic effects of this extracellular streptococcal product (3–5). As SLO has a prominent role in the pathogenesis of severe invasive infection in animal models (7), the recognition of SLO by HSA may have a significant protective role in human serum and sustains the emerging hypothesis that HSA is a player in the innate immunity system.

## Data Availability Statement

All datasets generated for this study are included in the article/**Supplementary Material**.

## Ethics Statement

Ethical review and approval was not required for the study. The patients/participants provided their written informed consent to participate in this study.

## Author Contributions

ADM, GDS, GMV, LL, and PA performed and analyzed the biochemical assays. ADM, DM, GDS, GMV, and LL performed the *in vitro* experiments. LL performed the bioinformatic analyses. ADM, AG, PA, RL, and SDB conceived and designed the experiments. ADM, FM, and SDB wrote the paper. All authors contributed to the article and approved the submitted version.

## Funding

This research was funded by the grant MAMBO (Ministero degli Affari Esteri e della Cooperazione Internazionale) to ADM and by Departments of Excellence - 2017 - legge 232/2016 - art.1, commi 314–337 awarded to the Department of Science, Roma Tre University, Rome, Italy for 2018-2022.

## Conflict of Interest

The authors declare that the research was conducted in the absence of any commercial or financial relationships that could be construed as a potential conflict of interest.

## Abbreviations

CDC, cholesterol-dependent cytolysin; CDI, *Clostridium difficile* infection; D4, domain 4; DTT, dithiothreitol; FA, fatty acid; FBS, fetal bovine serum; GAS, group A streptococcus; HEp-2, human epithelial type 2; HSA, human serum albumin; MTT, 3-(4,5-dimethylthiazol-2-yl)-2,5-diphenyltetrazolium bromide; PFT, pore-forming toxin; PAB, poly(A)-binding protein; PI, propidium iodide; RBC, red blood cells; SLO, streptolysin O; TcdA, *Clostridioides difficile* toxin A; TcdB, *Clostridioides difficile* toxin B.
