# Supplementary material for: Human Serum Albumin Binds Streptolysin O (SLO) Toxin Produced by Group A Streptococcus and Inhibits Its Cytotoxic and Hemolytic Effects
Source: Front Immunol. 2020 Dec 8;11:507092. doi: 10.3389/fimmu.2020.507092 (PMC7752801; doi:10.3389/fimmu.2020.507092)
Supplement: Supplementary file 1 [file DataSheet_1.docx]

***Supplementary Information***

**Human serum albumin binds streptolysin O (SLO) toxin produced by group A *Streptococcus* and inhibits its cytotoxic and hemolytic effects**

Gian Marco Vita^1,#^, Giovanna De Simone^1,#^, Loris Leboffe^1^, Francesca Montagnani^2,3^, Davide Mariotti^1^, Stefano Di Bella^4^, Roberto Luzzati^4^, Andrea Gori^5^,

Paolo Ascenzi^1^, Alessandra di Masi^1,*^

^1^ Department of Sciences, Roma Tre University, Roma, Italy

^2^ Department of Medical Biotechnologies, University of Siena, Siena, Italy

^3^ Department of Medical Sciences, Infectious and Tropical Diseases Unit, Hospital of Siena, Siena, Italy

^4^ University Hospital of Trieste, Trieste, Italy

^5^ Department of Pathophysiology and Transplantation, University of Milan, Milan, Italy

**Running title**: Human serum albumin prevents streptolysin O toxin pathogenicity

^#^ Co-first authors

**Corresponding author**: Alessandra di Masi, PhD, Department of Sciences, Roma Tre University, Viale G. Marconi 446, I-00146 Roma, Italy; tel: +39-06-57336363; fax: +39-06-57336321; e-mail: alessandra.dimasi@uniroma3.it

**Figure S1**

**A**

**
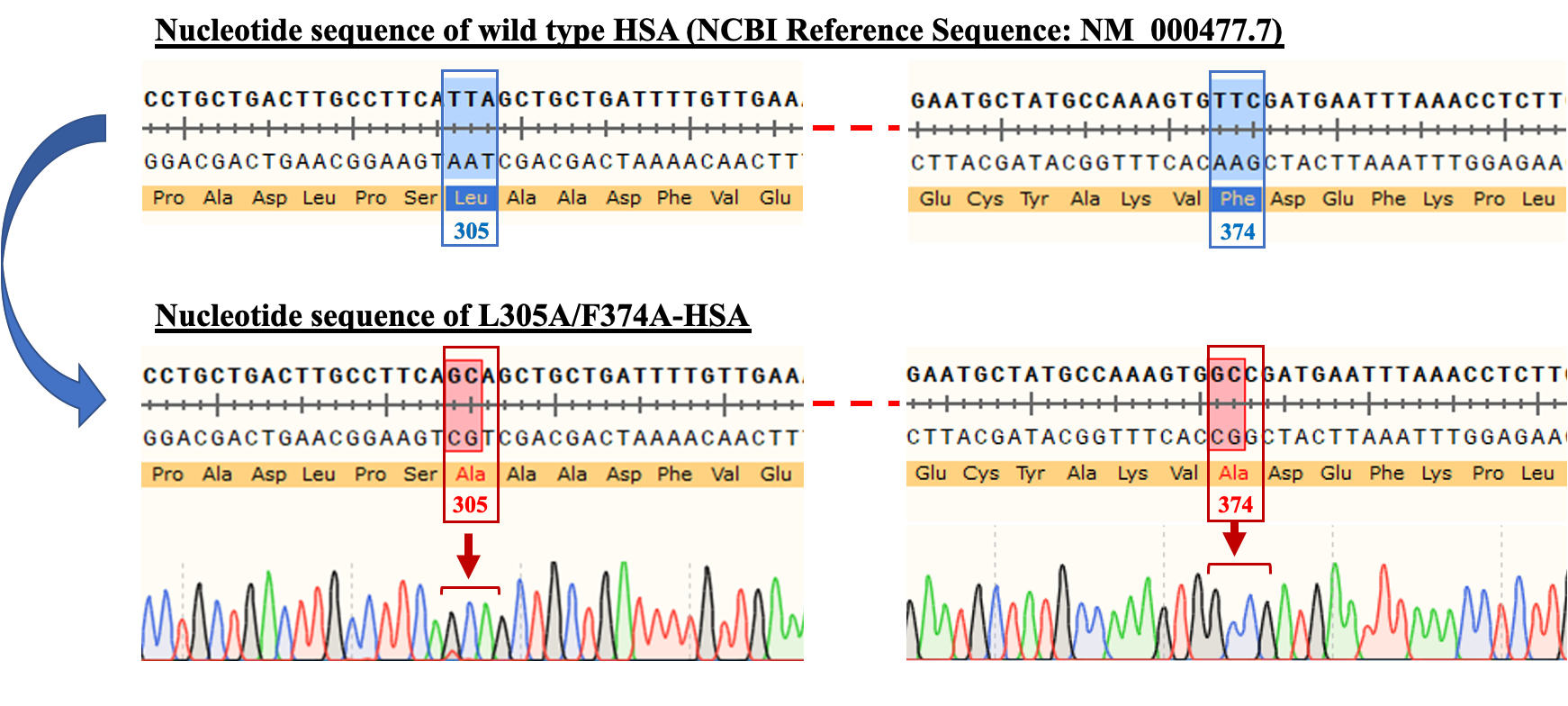
**

**B**

**
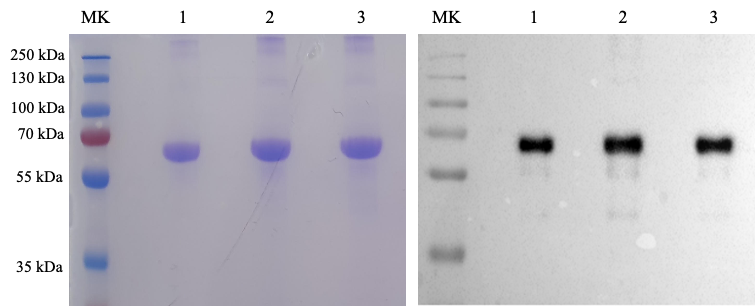
**

**Figure S1. Generation of recombinant HSA proteins. (**A) DNA sequencing of the double mutant L305A/F374A HSA. (B) Coomassie staining and Western blot analysis of recombinant 6×His-tag HSA. MK: marker; 1: 200 ng of commercial HSA; 2: 200 ng of recobinant wt-HSA; 3: 200 ng of recombinant L305A/F374A HSA. Commercial and recombinants HSA were detected by immunoblot using an anti-HSA antibody.

**Figure S2**

**Figure S2. Set-up of the best coating of ELISA plates with HSA.** Plates were coated with 100 μL of 2.0×10^-8^ M, 4.0×10^-8^ M, 8.0×10^-8^ M, and 1.6×10^-7^ M commercial HSA. The concentration of 1.6×10^-7^ M HSA allowed the saturation of the well. To evaluate wells coating, HSA was detected by the anti-HSA primary antibody. Readings were performed using TMB colorimetric substrate at 370 nm until reaction saturation. Results are representative of triplicates and are expressed as mean ± SD.

**Figure S3**

**
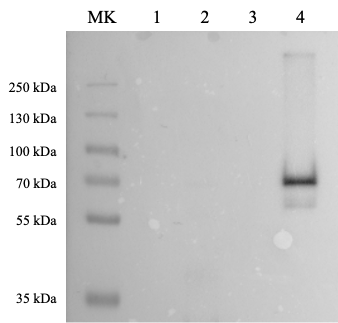
**

**Figure S3. Evaluation of anti-SLO antibody cross-reactivity.** Recombinant wt-HSA and SLO toxin were detected by Western blotting using the anti-SLO antibody. MK: marker; 1: Sample buffer 2x; 2: 200 ng recombinant wt-HSA; 3: Sample buffer 2x; 4: 160 ng SLO.

**Figure S4**

**
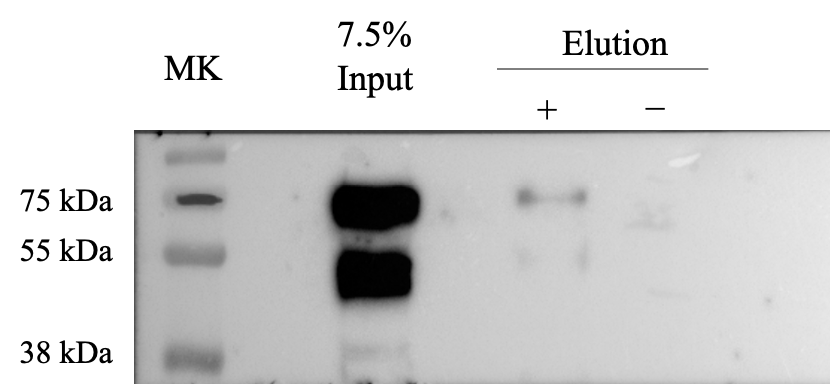
**

**Figure S4. Evaluation of the efficient binding of 4 μg recombinant wt-HSA to magnetic beads.** Thirty microliters of magnetic beads eluates were resolved on a 12.5% SDS-PAGE and HSA bound to the magnetic beads was detected by immunoblot using anti-HSA. MK: marker; (+) wt-HSA-conjugated beads; (−) unconjugated beads. The 7.5% of the total amount of wt-HSA was loaded as inputs.

**Figure S5**

******

**Figure S5. Evaluation of the cytotoxic effect of DTT in HEp-2 and A549 cells.** Cells were treated for 24 h with 2.0×10^−3^ M DTT. The percentage of viable cells was determined by MTT test, considering that control cells were taken as 100%. Data represent the mean value ± SD derived from three replicates.

**Figure S6**

**Figure S6. Evaluation of the potential HSA cytotoxicity in HEp-2 and A549 cells.** Confluent cell monolayers were cultured in serum-free medium for 24 h in the absence and presence of 1.0×10^‒5^ or 1.0×10^‒4^ M HSA. As control, cells were grown in complete medium (FBS). The percentage of viable cells was determined by MTT test, considering that control cells were taken as 100%. Data represent the mean value ± SD derived from three replicates.

**Figure S7**

**Figure S7. HSA does not induce red blood cells (RBCs) cell hemolysis.** RBCs, isolated from human whole blood of four healthy donors, and treated for 1 h at 37 °C with 1.0×10^−5^ M and 1.0×10^−4^ M HSA. As negative control, RBCs were incubated with PBS. As positive control, RBCs were disrupted with 2% Triton X-100. The concentration of released hemoglobin was quantiﬁed by measuring the absorbance at 541 nm. Results were represented as the percentage of lysed RBCs (assuming as 100% the positive control) derived from three independent experiments ± SD.
